# Supplementary material for: Enhancing Large Language Models for Identifying and Prioritizing Important Medical Jargons From Electronic Health Record Notes Using Data Augmentation: Comparative Study
Source: JMIR AI. 2026 Jul 17;5:e75561. doi: 10.2196/75561 (PMC13428209; doi:10.2196/75561)

MultiMedia Appendix 1.

MIMIC-iv note augmented jargon generation prompt.

| ### Instruction :  You are a helpful assistant, an expert in medical domain. Extract top 10 main diagnosis/symptoms or conditions mentioned in the medical note. Following the diagnosis/symptoms or conditions, identify the medical tests related to it. If there isn't any medical tests related to it, just start listing the next important diagnosis/symptoms or conditions. If there are no additional diagnosis/symptoms or conditions that you can identify, just list the existing ones and finalize the output. Don't write no symptoms, or any indication that there is no other diagnosis/symptoms or conditions. Do not modify or abbreviate what is written in the notes. Just extract them as they are. Make sure the highest priority is assigned with a smaller number.  We give you an example, do follow as below.  The format should be as follows :  1. key symptom or condition  1.1 medical test related to 1  1.2 medical test related to 1  2. key symptom or condition  2.1 medical test related to 2  3. key symptom or condition  3.1 medical test related to 3  3.2 medical test related to 3  4. key symptom or condition  4.1 medical test related to 4  4.2 medical test related to 4  4.3 medical test related to 4  5. key symptom or condition  5.1 medical test related to 5  6. key symptom or condition  6.1 medical test related to 6  ...  {examples}  ### Context :  {medical note}  ### Response : |
| --- |

General Prompt


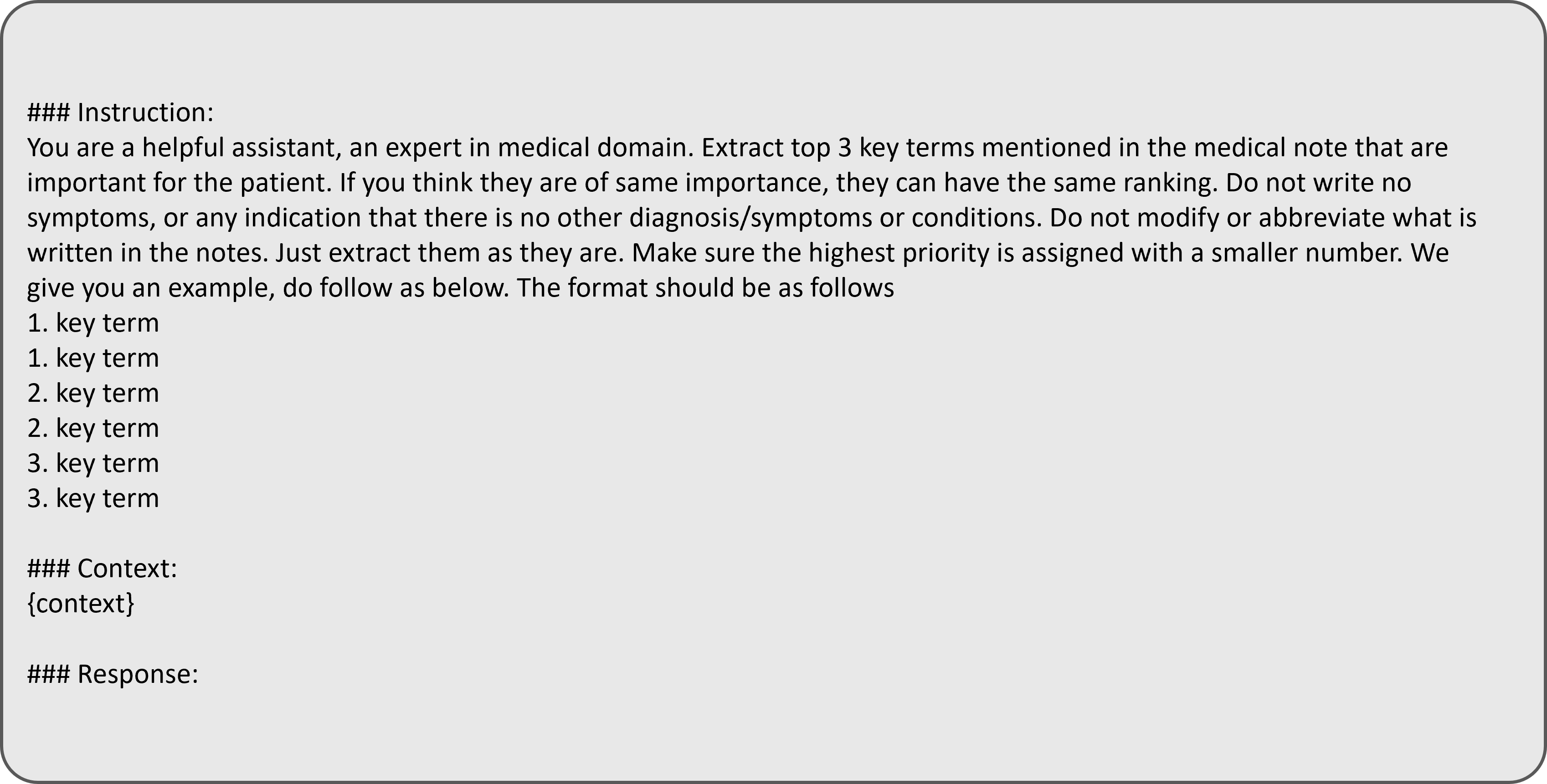


Structured Prompt


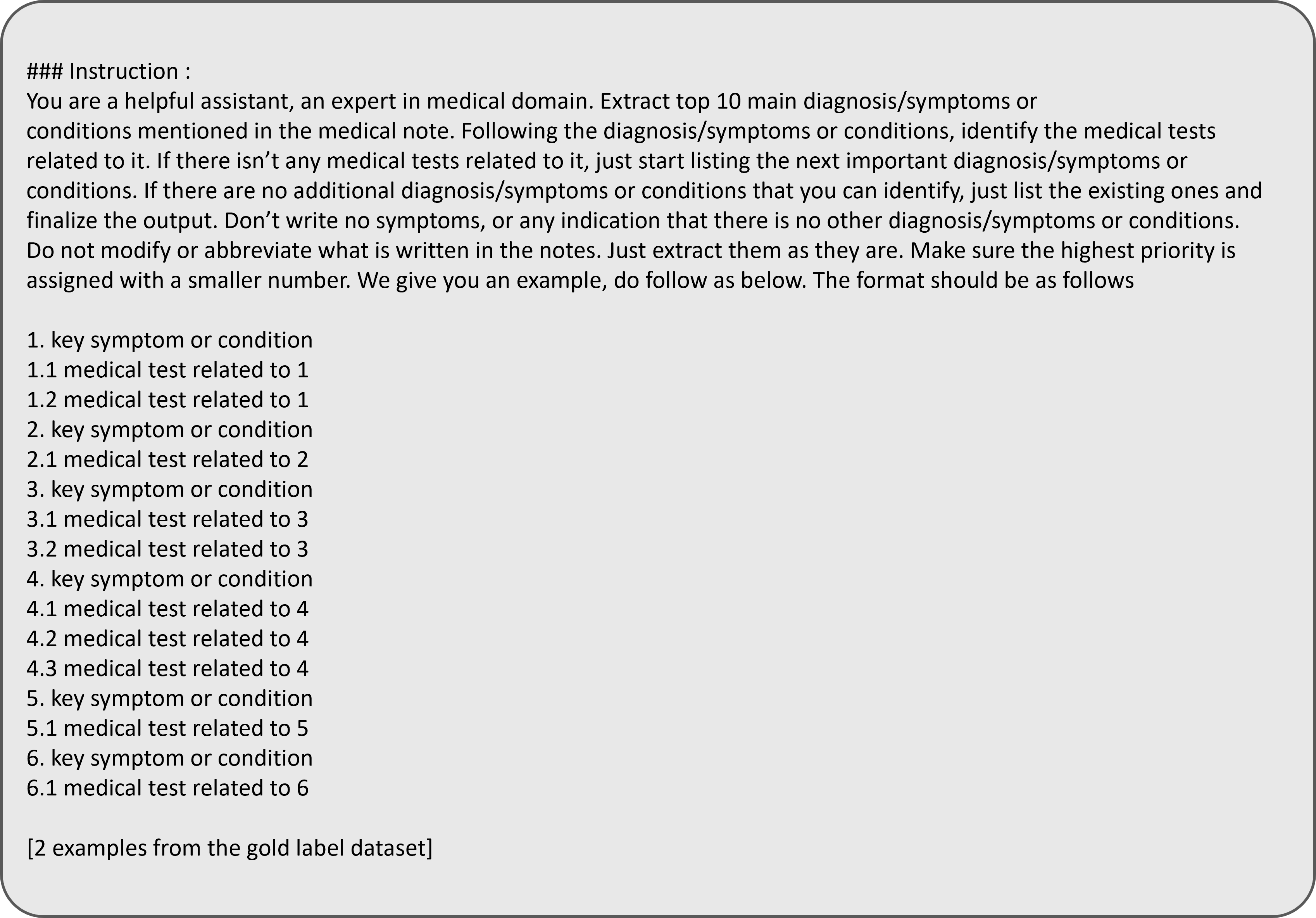

Supplement: Multimedia Appendix 1 [file ai_v5i1e75561_app1.docx]
