# Supplementary material for: Enhancing Large Language Models for Identifying and Prioritizing Important Medical Jargons From Electronic Health Record Notes Using Data Augmentation: Comparative Study
Source: JMIR AI. 2026 Jul 17;5:e75561. doi: 10.2196/75561 (PMC13428209; doi:10.2196/75561)
Supplement: Multimedia Appendix 2 [file ai_v5i1e75561_app2.docx]

MultiMedia Appendix 2. Supplementary results with Mean and Standard Deviation of Precision, Recall.

Supplementary Table 1A: Performance comparison between different models on zero-shot and few-shot tasks with top-5, and top-10 results for Precision (P) and Recall (R) and Standard Deviation (SD) in closed and open source LLMs using relaxed string matching.

| Model | Prompt | Zero-shot | | | | Few-shot | | | |
| --- | --- | --- | --- | --- | --- | --- | --- | --- | --- |
|  |  | 5 | | 10 | | 5 | | 10 | |
|  |  | P | R | P | R | P | R | P | R |
| Closed-Source | | | | | | | | | |
| GPT-5.2 | general | 0.477 (0.083) | 0.573 (0.088) | 0.465 (0.067) | 0.612 (0.087) | 0.444 (0.073) | 0.579 (0.097) | 0.45 (0.074) | 0.584  (0.1) |
|  | structured | 0.308 (0.103) | 0.452 (0.076) | 0.261 (0.079) | 0.595 (0.066) | 0.335 (0.102) | 0.473 (0.082) | 0.286 (0.086) | 0.623 (0.089) |
| GPT-5-mini | general | 0.546 (0.084) | 0.443 (0.081) | 0.473 (0.072) | 0.62 (0.094) | 0.495 (0.081) | 0.518 (0.105) | 0.465 (0.072) | 0.596 (0.075) |
|  | structured | 0.339 (0.075) | 0.55 (0.059) | 0.296 (0.076) | 0.704 (0.071) | 0.301 (0.083) | 0.498 (0.069) | 0.265 (0.081) | 0.615 (0.098) |
| Open-Source | | | | | | | | | |
| Mistral 7B | general | 0.436 (0.092) | 0.367 (0.096) | 0.38 (0.122) | 0.478 (0.13) | 0.464 (0.067) | 0.398 (0.069) | 0.46 (0.079) | 0.399 (0.071) |
|  | structured | 0.331 (0.07) | 0.457 (0.134) | 0.3  (0.073) | 0.544 (0.13) | 0.39 (0.087) | 0.425 (0.068) | 0.384  (0.1) | 0.426 (0.071) |
| Llama3.1-8B | general | 0.402 (0.066) | 0.343 (0.065) | 0.353 (0.068) | 0.45 (0.076) | 0.382 (0.091) | 0.467 (0.109) | 0.378 (0.089) | 0.477 (0.103) |
|  | structured | 0.389 (0.068) | 0.472  (0.1) | 0.367 (0.063) | 0.493 (0.112) | 0.357 (0.109) | 0.493 (0.135) | 0.363 (0.114) | 0.504 (0.151) |
| BioMistral 7B | general | 0.492 (0.134) | 0.144 (0.061) | 0.491 (0.135) | 0.146 (0.063) | 0.622 (0.113) | 0.139 (0.053) | 0.634 (0.122) | 0.138 (0.055) |
|  | structured | 0.745 (0.122) | 0.218 (0.105) | 0.745 (0.122) | 0.217 (0.107) | 0.611 (0.124) | 0.155 (0.071) | 0.611 (0.124) | 0.154 (0.071) |
| DeepSeek 8B | general | 0.462 (0.097) | 0.452 (0.08) | 0.39 (0.081) | 0.619 (0.075) | 0.28 (0.061) | 0.612 (0.131) | 0.263 (0.062) | 0.648 (0.125) |
|  | structured | 0.274 (0.046) | 0.486 (0.058) | 0.254 (0.055) | 0.586 (0.095) | 0.262 (0.071) | 0.564 (0.204) | 0.267 (0.071) | 0.579 (0.208) |

Supplementary Table 1B: Performance comparison between different models on zero-shot and few-shot tasks with top-5, and top-10 results for Precision (P) and Recall (R) and Standard Deviation (SD) in closed and open source LLMs using Jaccard Index.

| Model | Prompt | Zero-shot | | | | Few-shot | | | |
| --- | --- | --- | --- | --- | --- | --- | --- | --- | --- |
|  |  | 5 | | 10 | | 5 | | 10 | |
|  |  | P | R | P | R | P | R | P | R |
| Closed-Source | | | | | | | | | |
| GPT-5.2 | general | 0.296 (0.08) | 0.359 (0.08) | 0.291 (0.081) | 0.382 (0.097) | 0.307 (0.074) | 0.388 (0.062) | 0.311 (0.072) | 0.393 (0.06) |
|  | structured | 0.196 (0.069) | 0.282 (0.055) | 0.164 (0.048) | 0.376 (0.051) | 0.223 (0.077) | 0.309 (0.061) | 0.186 (0.058) | 0.406 (0.07) |
| GPT-5-mini | general | 0.16 (0.067) | 0.121 (0.047) | 0.142 (0.055) | 0.168 (0.048) | 0.254 (0.095) | 0.268 (0.099) | 0.236 (0.076) | 0.292 (0.077) |
|  | structured | 0.107 (0.05) | 0.172 (0.048) | 0.099 (0.045) | 0.228 (0.058) | 0.141 (0.047) | 0.224 (0.032) | 0.124 (0.045) | 0.288 (0.057) |
| Open-Source | | | | | | | | | |
| Mistral 7B | general | 0.384 (0.096) | 0.321 (0.094) | 0.33 (0.124) | 0.415 (0.121) | 0.406 (0.064) | 0.349 (0.066) | 0.406 (0.08) | 0.351 (0.068) |
|  | structured | 0.214 (0.051) | 0.341 (0.097) | 0.186 (0.048) | 0.414 (0.091) | 0.364 (0.082) | 0.391 (0.048) | 0.363 (0.096) | 0.392 (0.052) |
| Llama 3.1-8B | general | 0.37 (0.074) | 0.341 (0.069) | 0.325 (0.066) | 0.43 (0.083) | 0.351 (0.092) | 0.429 (0.134) | 0.349 (0.097) | 0.436 (0.131) |
|  | structured | 0.162 (0.043) | 0.276 (0.084) | 0.154 (0.044) | 0.299 (0.092) | 0.34 (0.099) | 0.464 (0.143) | 0.347 (0.102) | 0.473 (0.156) |
| BioMistral 7B | general | 0.044 (0.074) | 0.011 (0.021) | 0.044 (0.074) | 0.014 (0.03) | 0.355 (0.148) | 0.046 (0.021) | 0.366 (0.141) | 0.047 (0.019) |
|  | structured | 0.078 (0.071) | 0.01 (0.011) | 0.078 (0.071) | 0.01 (0.011) | 0.389 (0.167) | 0.058 (0.025) | 0.389 (0.167) | 0.057 (0.025) |
| DeepSeek 8B | general | 0.417 (0.073) | 0.419 (0.062) | 0.357 (0.073) | 0.581 (0.07) | 0.27 (0.059) | 0.606 (0.132) | 0.251 (0.058) | 0.642 (0.127) |
|  | structured | 0.175 (0.047) | 0.326 (0.097) | 0.163 (0.046) | 0.414 (0.115) | 0.229 (0.092) | 0.533 (0.212) | 0.233 (0.09) | 0.549 (0.213) |

Supplementary Table 2A. Performance with top-5, and top-10 results for Precision (P) and Recall (R) and Standard Deviation (SD) in fine-tuned LLMs using relaxed string matching.

| Model | 5 | | 10 | |
| --- | --- | --- | --- | --- |
|  | P | R | P | R |
| Mistral 7B | 0.451 (0.073) | 0.467 (0.097) | 0.363 (0.078) | 0.586 (0.12) |
| Llama 3.1 8B | 0.372 (0.063) | 0.53 (0.093) | 0.379 (0.07) | 0.532 (0.094) |
| BioMistral 7B | 0.387 (0.103) | 0.492 (0.074) | 0.393 (0.105) | 0.494 (0.071) |
| DeepSeek 8B | 0.409 (0.096) | 0.548 (0.074) | 0.426 (0.111) | 0.551 (0.07) |

Supplementary Table 2B. Performance with top-5, and top-10 results for Precision (P) and Recall (R) and Standard Deviation (SD) in fine-tuned LLMs using Jaccard Index.

| Model | 5 | | 10 | |
| --- | --- | --- | --- | --- |
|  | P | R | P | R |
| Mistral 7B | 0.467 (0.095) | 0.472 (0.08) | 0.472 (0.096) | 0.467 (0.081) |
| Llama 3.1 8B | 0.36 (0.066) | 0.558 (0.112) | 0.367 (0.072) | 0.562 (0.121) |
| BioMistral 7B | 0.384 (0.107) | 0.493 (0.081) | 0.391 (0.108) | 0.492 (0.077) |
| DeepSeek 8B | 0.407 (0.073) | 0.548 (0.098) | 0.418 (0.081) | 0.546 (0.098) |

Supplementary Table 3A. Performance with top-5, and top-10 results for Precision (P), Recall (R) F1 score (F1), MRR and Standard Deviation (SD) in MIMIC-IV fine-tuned models using relaxed string based matching.

| Model | Augmented Dataset Size (n) | 5 | | | | 10 | | | |
| --- | --- | --- | --- | --- | --- | --- | --- | --- | --- |
|  |  | P | R | F1 | MRR | P | R | F1 | MRR |
| Mistral 7B | 10 | 0.379 (0.06) | 0.33 (0.079) | 0.332 (0.061) | 0.536 (0.148) | 0.325 (0.057) | 0.412 (0.097) | 0.337 (0.058) | 0.527 (0.146) |
|  | 100 | 0.445 (0.073) | 0.427 (0.077) | 0.408 (0.058) | 0.514 (0.152) | 0.399 (0.061) | 0.483 (0.063) | 0.407 (0.05) | 0.508 (0.15) |
|  | 1000 | 0.388 (0.1) | 0.414 (0.066) | 0.374 (0.078) | 0.544 (0.176) | 0.362 (0.073) | 0.514 (0.082) | 0.398 (0.075) | 0.534 (0.169) |
|  | 10000 | 0.396 (0.081) | 0.416 (0.058) | 0.382 (0.061) | 0.568 (0.158) | 0.379 (0.059) | 0.525 (0.065) | 0.413 (0.055) | 0.564 (0.154) |
| Llama 3.1 8B | 10 | 0.368 (0.074) | 0.321 (0.064) | 0.318 (0.057) | 0.418 (0.104) | 0.344 (0.059) | 0.422 (0.082) | 0.349 (0.055) | 0.399 (0.101) |
|  | 100 | 0.394 (0.062) | 0.397 (0.056) | 0.372 (0.047) | 0.534 (0.165) | 0.371 (0.038) | 0.467 (0.059) | 0.385 (0.034) | 0.524 (0.162) |
|  | 1000 | 0.393 (0.059) | 0.413 (0.067) | 0.375 (0.047) | 0.537 (0.164) | 0.367 (0.053) | 0.468 (0.059) | 0.383 (0.045) | 0.536 (0.166) |
|  | 10000 | 0.394 (0.083) | 0.404 (0.062) | 0.375 (0.067) | 0.555 (0.158) | 0.39 (0.075) | 0.499 (0.082) | 0.408 (0.075) | 0.546 (0.146) |
| BioMistral 7B | 10 | 0.37 (0.044) | 0.27 (0.051) | 0.294 (0.049) | 0.46 (0.169) | 0.338 (0.05) | 0.334 (0.061) | 0.312 (0.056) | 0.451 (0.167) |
|  | 100 | 0.35 (0.049) | 0.401 (0.057) | 0.351 (0.043) | 0.489 (0.146) | 0.346 (0.048) | 0.448 (0.065) | 0.363 (0.044) | 0.478 (0.149) |
|  | 1000 | 0.405 (0.084) | 0.429 (0.066) | 0.387 (0.064) | 0.575 (0.125) | 0.38 (0.076) | 0.482 (0.07) | 0.393 (0.063) | 0.566 (0.112) |
|  | 10000 | 0.409 (0.072) | 0.432 (0.059) | 0.391 (0.053) | 0.527 (0.137) | 0.383 (0.056) | 0.512 (0.071) | 0.405 (0.054) | 0.503 (0.116) |
| DeepSeek 8B | 10 | 0.438 (0.078) | 0.403 (0.058) | 0.391 (0.052) | 0.482 (0.133) | 0.383 (0.064) | 0.52 (0.051) | 0.411 (0.045) | 0.483 (0.133) |
|  | 100 | 0.367 (0.068) | 0.46 (0.097) | 0.378 (0.064) | 0.499 (0.163) | 0.352 (0.065) | 0.542 (0.107) | 0.396 (0.069) | 0.499 (0.163) |
|  | 1000 | 0.389 (0.087) | 0.459 (0.084) | 0.391 (0.073) | 0.522 (0.133) | 0.362 (0.071) | 0.541 (0.099) | 0.408 (0.072) | 0.512 (0.131) |
|  | 10000 | 0.413 (0.065) | 0.441 (0.059) | 0.398 (0.044) | **0.581 (0.105)** | 0.399 (0.061) | 0.53 (0.065) | **0.425 (0.052)** | 0.572 (0.105) |

Supplementary Table 3B. Performance with top-5, and top-10 results for Precision (P), Recall (R) F1 score (F1), MRR and Standard Deviation (SD) in MIMIC-IV fine-tuned models using Jaccard Index.

| Model | Augmented Dataset Size (n) | 5 | | | | 10 | | | |
| --- | --- | --- | --- | --- | --- | --- | --- | --- | --- |
|  |  | P | R | F1 | MRR | P | R | F1 | MRR |
| Mistral 7B | 10 | 0.312 (0.058) | 0.277 (0.058) | 0.276 (0.046) | 0.494 (0.109) | 0.264 (0.059) | 0.342 (0.069) | 0.276 (0.052) | 0.485 (0.115) |
|  | 100 | 0.373 (0.054) | 0.367 (0.058) | 0.347 (0.045) | 0.465 (0.128) | 0.338 (0.044) | 0.417 (0.057) | 0.347 (0.045) | 0.455 (0.12) |
|  | 1000 | 0.371 (0.099) | 0.395 (0.065) | 0.356 (0.078) | 0.528 (0.107) | 0.343 (0.069) | 0.479 (0.075) | 0.375 (0.07) | 0.528 (0.107) |
|  | 10000 | 0.376 (0.079) | 0.391 (0.057) | 0.361 (0.06) | 0.561 (0.149) | 0.36 (0.054) | 0.49 (0.059) | 0.39 (0.051) | 0.557 (0.145) |
| Llama 3.1 8B | 10 | 0.276 (0.062) | 0.242 (0.061) | 0.239 (0.05) | 0.322 (0.075) | 0.258 (0.063) | 0.311 (0.06) | 0.259 (0.048) | 0.312 (0.086) |
|  | 100 | 0.344 (0.053) | 0.345 (0.054) | 0.324 (0.046) | 0.47 (0.119) | 0.325 (0.034) | 0.397 (0.058) | 0.332 (0.037) | 0.469 (0.119) |
|  | 1000 | 0.374 (0.061) | 0.391 (0.066) | 0.356 (0.049) | 0.503 (0.142) | 0.35 (0.052) | 0.442 (0.062) | 0.364 (0.046) | 0.502 (0.144) |
|  | 10000 | 0.378 (0.074) | 0.385 (0.051) | 0.358 (0.058) | 0.549 (0.148) | 0.374 (0.062) | 0.471 (0.068) | 0.389 (0.062) | 0.539 (0.136) |
| BioMistral 7B | 10 | 0.266 (0.091) | 0.199 (0.057) | 0.212 (0.068) | 0.357 (0.143) | 0.24 (0.059) | 0.241 (0.052) | 0.223 (0.051) | 0.357 (0.143) |
|  | 100 | 0.315 (0.046) | 0.356 (0.051) | 0.314 (0.043) | 0.431 (0.14) | 0.312 (0.043) | 0.401 (0.062) | 0.326 (0.045) | 0.431 (0.143) |
|  | 1000 | 0.356 (0.059) | 0.373 (0.046) | 0.337 (0.039) | 0.541 (0.106) | 0.338 (0.054) | 0.421 (0.048) | 0.346 (0.039) | 0.543 (0.108) |
|  | 10000 | 0.383 (0.071) | 0.397 (0.068) | 0.364 (0.056) | 0.495 (0.103) | 0.356 (0.052) | 0.461 (0.07) | 0.372 (0.046) | 0.476 (0.085) |
| DeepSeek 8B | 10 | 0.368 (0.08) | 0.352 (0.082) | 0.332 (0.073) | 0.419 (0.178) | 0.316 (0.07) | 0.439 (0.082) | 0.341 (0.065) | 0.421 (0.176) |
|  | 100 | 0.322 (0.05) | 0.409 (0.085) | 0.333 (0.049) | 0.457 (0.17) | 0.306 (0.045) | 0.468 (0.088) | 0.343 (0.05) | 0.457 (0.17) |
|  | 1000 | 0.373 (0.083) | 0.436 (0.083) | 0.374 (0.07) | 0.518 (0.103) | 0.35 (0.068) | 0.516 (0.094) | 0.391 (0.071) | 0.519 (0.102) |
|  | 10000 | 0.391 (0.049) | 0.416 (0.045) | 0.376 (0.024) | 0.575 (0.103) | 0.377 (0.043) | 0.499 (0.054) | 0.401 (0.038) | 0.565 (0.104) |

Supplementary Table 4A. Precision (P) and Recall (R) for Baseline models using relaxed string matching.

| Model | P | R |
| --- | --- | --- |
| MedJEx | 0.067 (0.016) | 0.726 (0.059) |
| BioClinical- ModernBERT | 0.104 (0.093) | 0.085 (0.091) |

Supplementary Table 4B. Precision (P) and Recall (R) for Baseline models Jaccard Index.

| Model | P | R |
| --- | --- | --- |
| MedJEx | 0.077 (0.019) | 0.824 (0.079) |
| BioClinical- ModernBERT | 0.121 (0.019) | 0.094 (0.095) |

Supplementary Table 5. F1 and MRR for vanilla open/closed source models based on disease category using relaxed string matching.

|  |  |  |  | zeroshot | | fewshot | |
| --- | --- | --- | --- | --- | --- | --- | --- |
| Model | Prompt | Disease | N | F1 | MRR | F1 | MRR |
| GPT-5.2 | general | Cancer | 17 | 0.484 (0.194) | 0.505 (0.342) | 0.491 (0.184) | 0.532 (0.281) |
|  |  | Copd | 20 | 0.479 (0.186) | 0.595 (0.374) | 0.467 (0.195) | 0.485 (0.313) |
|  |  | Diabetes | 15 | 0.519 (0.170) | 0.648 (0.373) | 0.479 (0.180) | 0.722 (0.337) |
|  |  | Heart Failure | 18 | 0.483 (0.227) | 0.606 (0.246) | 0.455 (0.196) | 0.520 (0.258) |
|  |  | Hypertension | 11 | 0.474 (0.139) | 0.530 (0.371) | 0.488 (0.143) | 0.630 (0.357) |
|  |  | Liver Failure | 9 | 0.568 (0.186) | 0.563 (0.318) | 0.506 (0.191) | 0.515 (0.315) |
|  | structured | Cancer | 17 | 0.338 (0.167) | 0.526 (0.393) | 0.372 (0.210) | 0.511 (0.393) |
|  |  | Copd | 20 | 0.354 (0.203) | 0.463 (0.423) | 0.364 (0.163) | 0.543 (0.360) |
|  |  | Diabetes | 15 | 0.280 (0.156) | 0.533 (0.446) | 0.301 (0.149) | 0.499 (0.451) |
|  |  | Heart Failure | 18 | 0.338 (0.129) | 0.451 (0.416) | 0.371 (0.159) | 0.567 (0.411) |
|  |  | Hypertension | 11 | 0.356 (0.115) | 0.515 (0.398) | 0.357 (0.113) | 0.455 (0.422) |
|  |  | Liver Failure | 9 | 0.356 (0.170) | 0.685 (0.429) | 0.446 (0.191) | 0.667 (0.382) |
| GPT-5-mini | general | Cancer | 17 | 0.537 (0.203) | 0.585 (0.384) | 0.463 (0.200) | 0.607 (0.327) |
|  |  | Copd | 20 | 0.505 (0.167) | 0.499 (0.375) | 0.498 (0.164) | 0.495 (0.329) |
|  |  | Diabetes | 15 | 0.510 (0.218) | 0.482 (0.448) | 0.507 (0.177) | 0.585 (0.426) |
|  |  | Heart Failure | 18 | 0.468 (0.183) | 0.574 (0.384) | 0.483 (0.200) | 0.545 (0.314) |
|  |  | Hypertension | 11 | 0.460 (0.194) | 0.545 (0.395) | 0.517 (0.186) | 0.445 (0.358) |
|  |  | Liver Failure | 9 | 0.563 (0.237) | 0.685 (0.429) | 0.498 (0.215) | 0.574 (0.465) |
|  | structured | Cancer | 17 | 0.435 (0.220) | 0.452 (0.336) | 0.360 (0.244) | 0.376 (0.350) |
|  |  | Copd | 20 | 0.391 (0.176) | 0.590 (0.391) | 0.339 (0.187) | 0.477 (0.405) |
|  |  | Diabetes | 15 | 0.339 (0.187) | 0.499 (0.415) | 0.316 (0.165) | 0.459 (0.475) |
|  |  | Heart Failure | 18 | 0.383 (0.148) | 0.618 (0.393) | 0.344 (0.135) | 0.479 (0.436) |
|  |  | Hypertension | 11 | 0.355 (0.128) | 0.576 (0.360) | 0.368 (0.122) | 0.538 (0.371) |
|  |  | Liver Failure | 9 | 0.417 (0.186) | 0.722 (0.441) | 0.376 (0.168) | 0.796 (0.351) |
| Llama 3.1-8B | general | Cancer | 17 | 0.349 (0.371) | 0.269 (0.339) | 0.292 (0.270) | 0.461 (0.384) |
|  |  | Copd | 20 | 0.360 (0.242) | 0.467 (0.461) | 0.420 (0.240) | 0.426 (0.377) |
|  |  | Diabetes | 15 | 0.344 (0.323) | 0.322 (0.386) | 0.369 (0.247) | 0.665 (0.348) |
|  |  | Heart Failure | 18 | 0.378 (0.245) | 0.542 (0.430) | 0.358 (0.244) | 0.471 (0.403) |
|  |  | Hypertension | 11 | 0.475 (0.278) | 0.561 (0.396) | 0.412 (0.261) | 0.642 (0.326) |
|  |  | Liver Failure | 9 | 0.314 (0.184) | 0.370 (0.423) | 0.369 (0.208) | 0.556 (0.333) |
|  | structured | Cancer | 17 | 0.382 (0.278) | 0.488 (0.327) | 0.360 (0.276) | 0.402 (0.350) |
|  |  | Copd | 20 | 0.332 (0.207) | 0.549 (0.410) | 0.366 (0.270) | 0.431 (0.417) |
|  |  | Diabetes | 15 | 0.293 (0.211) | 0.444 (0.452) | 0.358 (0.301) | 0.422 (0.358) |
|  |  | Heart Failure | 18 | 0.334 (0.201) | 0.544 (0.411) | 0.401 (0.241) | 0.470 (0.384) |
|  |  | Hypertension | 11 | 0.414 (0.321) | 0.455 (0.422) | 0.346 (0.313) | 0.553 (0.384) |
|  |  | Liver Failure | 9 | 0.455 (0.203) | 0.643 (0.289) | 0.424 (0.270) | 0.383 (0.468) |
| Mistral 7B | general | Cancer | 17 | 0.334 (0.282) | 0.386 (0.414) | 0.364 (0.282) | 0.401 (0.370) |
|  |  | Copd | 20 | 0.423 (0.233) | 0.358 (0.406) | 0.375 (0.216) | 0.442 (0.413) |
|  |  | Diabetes | 15 | 0.401 (0.323) | 0.393 (0.438) | 0.361 (0.247) | 0.480 (0.348) |
|  |  | Heart Failure | 18 | 0.419 (0.300) | 0.456 (0.451) | 0.397 (0.193) | 0.620 (0.366) |
|  |  | Hypertension | 11 | 0.529 (0.292) | 0.614 (0.388) | 0.459 (0.160) | 0.530 (0.420) |
|  |  | Liver Failure | 9 | 0.289 (0.235) | 0.481 (0.503) | 0.350 (0.159) | 0.556 (0.400) |
|  | structured | Cancer | 17 | 0.364 (0.233) | 0.485 (0.301) | 0.332 (0.225) | 0.440 (0.352) |
|  |  | Copd | 20 | 0.314 (0.209) | 0.525 (0.373) | 0.359 (0.224) | 0.473 (0.416) |
|  |  | Diabetes | 15 | 0.381 (0.242) | 0.583 (0.407) | 0.292 (0.193) | 0.658 (0.301) |
|  |  | Heart Failure | 18 | 0.272 (0.164) | 0.674 (0.446) | 0.434 (0.186) | 0.610 (0.403) |
|  |  | Hypertension | 11 | 0.432 (0.181) | 0.595 (0.326) | 0.368 (0.204) | 0.597 (0.315) |
|  |  | Liver Failure | 9 | 0.395 (0.284) | 0.597 (0.403) | 0.334 (0.274) | 0.407 (0.345) |
| BioMistral 7B | general | Cancer | 17 | 0.230 (0.308) | 0.311 (0.416) | 0.169 (0.257) | 0.494 (0.501) |
|  |  | Copd | 20 | 0.195 (0.242) | 0.375 (0.483) | 0.230 (0.269) | 0.375 (0.483) |
|  |  | Diabetes | 15 | 0.162 (0.248) | 0.278 (0.426) | 0.165 (0.161) | 0.449 (0.465) |
|  |  | Heart Failure | 18 | 0.240 (0.299) | 0.407 (0.493) | 0.223 (0.182) | 0.537 (0.500) |
|  |  | Hypertension | 11 | 0.207 (0.246) | 0.258 (0.375) | 0.309 (0.254) | 0.485 (0.480) |
|  |  | Liver Failure | 9 | 0.235 (0.183) | 0.519 (0.503) | 0.143 (0.227) | 0.407 (0.494) |
|  | structured | Cancer | 17 | 0.315 (0.278) | 0.596 (0.395) | 0.209 (0.259) | 0.510 (0.502) |
|  |  | Copd | 20 | 0.318 (0.289) | 0.587 (0.468) | 0.337 (0.292) | 0.432 (0.443) |
|  |  | Diabetes | 15 | 0.184 (0.213) | 0.389 (0.453) | 0.200 (0.248) | 0.344 (0.456) |
|  |  | Heart Failure | 18 | 0.283 (0.242) | 0.448 (0.484) | 0.174 (0.146) | 0.389 (0.502) |
|  |  | Hypertension | 11 | 0.352 (0.338) | 0.487 (0.456) | 0.239 (0.288) | 0.515 (0.503) |
|  |  | Liver Failure | 9 | 0.434 (0.320) | 0.500 (0.500) | 0.136 (0.173) | 0.444 (0.527) |
| DeepSeek 8B | general | Cancer | 17 | 0.431 (0.189) | 0.394 (0.416) | 0.337 (0.212) | 0.406 (0.324) |
|  |  | Copd | 20 | 0.431 (0.230) | 0.365 (0.357) | 0.335 (0.233) | 0.473 (0.334) |
|  |  | Diabetes | 15 | 0.412 (0.235) | 0.217 (0.292) | 0.385 (0.211) | 0.607 (0.339) |
|  |  | Heart Failure | 18 | 0.468 (0.201) | 0.543 (0.346) | 0.381 (0.201) | 0.473 (0.289) |
|  |  | Hypertension | 11 | 0.496 (0.138) | 0.506 (0.451) | 0.281 (0.183) | 0.405 (0.329) |
|  |  | Liver Failure | 9 | 0.432 (0.249) | 0.519 (0.412) | 0.318 (0.155) | 0.574 (0.324) |
|  | structured | Cancer | 17 | 0.348 (0.193) | 0.480 (0.353) | 0.361 (0.266) | 0.511 (0.383) |
|  |  | Copd | 20 | 0.297 (0.186) | 0.454 (0.388) | 0.310 (0.254) | 0.419 (0.385) |
|  |  | Diabetes | 15 | 0.359 (0.174) | 0.428 (0.428) | 0.309 (0.214) | 0.428 (0.382) |
|  |  | Heart Failure | 18 | 0.272 (0.192) | 0.569 (0.426) | 0.320 (0.173) | 0.512 (0.346) |
|  |  | Hypertension | 11 | 0.436 (0.165) | 0.402 (0.385) | 0.382 (0.158) | 0.514 (0.311) |
|  |  | Liver Failure | 9 | 0.286 (0.255) | 0.417 (0.468) | 0.331 (0.269) | 0.541 (0.280) |

Supplementary Table 6. F1 and MRR for fine-tuned models based on disease category.

| Model | Disease | N | F1 | MRR |
| --- | --- | --- | --- | --- |
| Llama 3.1 8B | Cancer | 17 | 0.360 (0.220) | 0.582 (0.280) |
|  | Copd | 20 | 0.398 (0.241) | 0.476 (0.321) |
|  | Diabetes | 15 | 0.388 (0.183) | 0.495 (0.237) |
|  | Heart Failure | 18 | 0.396 (0.177) | 0.383 (0.280) |
|  | Hypertension | 11 | 0.387 (0.165) | 0.542 (0.279) |
|  | Liver Failure | 9 | 0.364 (0.158) | 0.741 (0.252) |
| Mistral 7B | Cancer | 17 | 0.385 (0.234) | 0.528 (0.356) |
|  | Copd | 20 | 0.450 (0.232) | 0.548 (0.351) |
|  | Diabetes | 15 | 0.441 (0.179) | 0.549 (0.362) |
|  | Heart Failure | 18 | 0.374 (0.183) | 0.481 (0.354) |
|  | Hypertension | 11 | 0.501 (0.162) | 0.567 (0.287) |
|  | Liver Failure | 9 | 0.335 (0.209) | 0.463 (0.398) |
| BioMistral 7B | Cancer | 17 | 0.403 (0.206) | 0.472 (0.276) |
|  | Copd | 20 | 0.374 (0.152) | 0.573 (0.364) |
|  | Diabetes | 15 | 0.383 (0.138) | 0.521 (0.387) |
|  | Heart Failure | 18 | 0.446 (0.237) | 0.665 (0.334) |
|  | Hypertension | 11 | 0.300 (0.174) | 0.718 (0.359) |
|  | Liver Failure | 9 | 0.276 (0.150) | 0.410 (0.401) |
| DeepSeek 8B | Cancer | 17 | 0.503 (0.264) | 0.416 (0.349) |
|  | Copd | 20 | 0.408 (0.214) | 0.535 (0.397) |
|  | Diabetes | 15 | 0.458 (0.251) | 0.434 (0.343) |
|  | Heart Failure | 18 | 0.394 (0.203) | 0.633 (0.221) |
|  | Hypertension | 11 | 0.404 (0.213) | 0.491 (0.326) |
|  | Liver Failure | 9 | 0.336 (0.267) | 0.509 (0.294) |

Supplementary Table 7. F1 and MRR for MIMIC-IV fine-tuned models based on disease category.

| Model | Dataset Size | Disease | N | F1 | MRR |
| --- | --- | --- | --- | --- | --- |
| Llama 3.1-8B | 10000 | Cancer | 17 | 0.390 (0.181) | 0.471 (0.404) |
|  |  | Copd | 20 | 0.418 (0.191) | 0.390 (0.406) |
|  |  | Diabetes | 15 | 0.376 (0.175) | 0.489 (0.455) |
|  |  | Heart Failure | 18 | 0.421 (0.169) | 0.763 (0.389) |
|  |  | Hypertension | 11 | 0.422 (0.163) | 0.597 (0.440) |
|  |  | Liver Failure | 9 | 0.428 (0.141) | 0.630 (0.455) |
| Mistral 7B | 10000 | Cancer | 17 | 0.398 (0.164) | 0.553 (0.421) |
|  |  | Copd | 20 | 0.440 (0.215) | 0.434 (0.401) |
|  |  | Diabetes | 15 | 0.398 (0.195) | 0.383 (0.432) |
|  |  | Heart Failure | 18 | 0.392 (0.173) | 0.763 (0.389) |
|  |  | Hypertension | 11 | 0.460 (0.179) | 0.597 (0.379) |
|  |  | Liver Failure | 9 | 0.393 (0.120) | 0.741 (0.401) |
| BioMistral 7B | 10000 | Cancer | 17 | 0.428 (0.148) | 0.523 (0.412) |
|  |  | Copd | 20 | 0.416 (0.216) | 0.432 (0.387) |
|  |  | Diabetes | 15 | 0.353 (0.119) | 0.467 (0.422) |
|  |  | Heart Failure | 18 | 0.403 (0.177) | 0.551 (0.412) |
|  |  | Hypertension | 11 | 0.443 (0.164) | 0.542 (0.422) |
|  |  | Liver Failure | 9 | 0.380 (0.065) | 0.537 (0.455) |
| DeepSeek 8B | 10000 | Cancer | 17 | 0.453 (0.179) | 0.450 (0.411) |
|  |  | Copd | 20 | 0.428 (0.204) | 0.495 (0.429) |
|  |  | Diabetes | 15 | 0.388 (0.188) | 0.489 (0.455) |
|  |  | Heart Failure | 18 | 0.449 (0.181) | 0.698 (0.426) |
|  |  | Hypertension | 11 | 0.466 (0.138) | 0.665 (0.361) |
|  |  | Liver Failure | 9 | 0.327 (0.118) | 0.741 (0.401) |
